# Weight, weight gain and behavioural risk factors in women attending a breast cancer family history, risk and prevention clinic: an observational study

**DOI:** 10.1038/s44276-024-00039-9

**Published:** 2024-03-14

**Authors:** Mary Pegington, John Belcher, Emma Barrett, Pawandeep Virpal, Anthony Howell, D. Gareth Evans, Michelle Harvie

**Affiliations:** 1 Division of Cancer Sciences, School of Medical Sciences, Faculty of Biology, Medicine and Health, The University of Manchester, Wilmslow Road, Manchester, M20 4BX UK; 2 The Prevent Breast Cancer Research Unit, The Nightingale Centre, Manchester University NHS Foundation Trust, Manchester, M23 9LT UK; 3 School of Medicine, Keele University, Keele, Staffordshire ST5 5BG UK; 4 Research and Innovation Division, Manchester University NHS Foundation Trust, Manchester, M23 9LT UK; 5 Manchester Breast Centre, Manchester Cancer Research Centre, University of Manchester, 555 Wilmslow Rd, Manchester, M20 4GJ UK; 6 Department of Medical Oncology, The Christie NHS Foundation Trust, Wilmslow Rd, Manchester, M20 4BX UK; 7 NW Genomic Laboratory Hub, Manchester Centre for Genomic Medicine, Manchester University Hospitals NHS Foundation Trust, Manchester, UK; 8 Genomic Medicine, Division of Evolution and Genomic Sciences, The University of Manchester, St Mary’s Hospital, Manchester University NHS Foundation Trust, Oxford Road, Manchester, M13 9WL UK

## Abstract

**Background:**

Weight and health behaviours impact on breast cancer risk. We describe trends in weight and health behaviours in women at entry to a specialist breast cancer family history clinic in Manchester, UK, and changes after clinic entry.

**Methods:**

Questionnaires were completed at clinic entry (1987–2019, *n* = 10,920), and updated in 2010–11 (*n* = 3283). Clinic entry characteristics were compared between joining periods 1989–98, 1999–2008 and 2009–18. Partial Least Squares analysis characterised trends in weight, smoking and alcohol intake by age at entry, year of entry and birth year. Weight changes were compared over time, between joining periods.

**Results:**

Obesity at clinic entry increased from 10.6% in 1989–98 to 20.5% in 2009–18. Alcohol intake above recommendations and smoking prevalence decreased from 20.1% to 13.8% and 33.5% to 16.1% respectively. Weight gain was median 9.7 (IQR 1.4–20.6) % between age 20 and clinic entry (mean duration 11.9 ± 5.6 years) and a further 4.5 (0.0–12.5) % between clinic entry and 2010–11 (31.1 ± 10.4 years). Weight gain between age 20 and clinic entry was highest in the most recent joining period.

**Conclusions:**

Obesity and weight gain are common in women attending a breast cancer family history clinic suggesting a need for weight management advice and support.

## Background

Breast cancer (BC) is the most common cancer in the UK with 55,109 diagnoses and 11,371 deaths in 2017 [1] and incidence is predicted to increase [2]. Weight and health behaviours play an important role in determining risk of BC. Published estimates have reported 8% of UK cases are associated with overweight/obesity which increases the risk of postmenopausal BC, and another 8% with alcohol which increases both pre- and postmenopausal risk [3]. Gaining weight during adulthood (from age 18–20 years) increases risk of postmenopausal BC risk by 6% per 5 kg (RR 1.06, 95% confidence interval [CI] 1.05–1.08) [4]. Other health behaviours associated with increased risk are lack of physical activity and smoking [4, 5].

A recent report highlighted that approximately 40% of BC cases occur in the 20% of the women who are at increased risk of BC (≥16.7% or 1 in 6 lifetime risk) [6, 7]. Evidence suggests being a healthy weight and adhering to healthy behaviours is beneficial for this increased risk population for reducing both BC risk [8–10] and overall mortality [11].

Many women at increased risk attend Family History, Risk and Prevention Clinics (FHRPCs) in the UK for BC risk assessment, mammography, advice on risk-reducing medication and preventative surgery. Women are referred from primary care into FHRPCs according to NICE Clinical Guideline 164 regarding familial breast cancer, for example if they have one first-degree female relative diagnosed with BC at younger than age 40 years [12]. The FHRPC at Manchester University NHS Foundation Trust in North West England has received over 14,000 referrals since opening in 1987, and has been described previously [13]. Attendees receive standard leaflets describing links between weight, health behaviours and BC risk which may be discussed with clinicians, but individualised health behaviour advice and support are not provided.

The aim of the current study was to:

1. Describe trends in weight, body mass index (BMI) and behavioural risk factors amongst women at the time of entry to the Manchester FHRPC over the last three decades and whether this varies according to age of joining the clinic, year of joining the clinic, or birth year.
2. Describe changes in weight and BMI:
  - after joining the FHRPC
  - during adulthood from age 20
3. Describe changes in alcohol consumption after joining the FHRPC.

We hypothesised that our analysis would show an increase in weight and BMI, and a reduction in smoking prevalence at FHRPC entry over the last three decades, all of which have been observed in the general UK female, adult population [14]. We hypothesised no significant change to alcohol intake at FHRPC entry due to the mixed picture of alcohol consumption over the last three decades [15–17]. We also hypothesised increases in weight and BMI in women after age 20 and after joining the FHRPC, and no significant change to alcohol consumption.

## Methods

This paper is prepared according to STROBE guidelines on reporting cohort studies detailed in Supplementary Table 1 [18].

### Data sources

Data from two questionnaires were used. All women complete an FHRPC entry questionnaire upon joining the clinic, including postcode, date of birth, self-reported weight and height, smoking history and current alcoholic drinks per week (Supplementary Fig. 1). From 2017 this questionnaire also included self-reported weight at age 20 years. BC risk estimation at the time of joining clinic was based on a modification of the Claus tables until 2003 and the Tyrer-Cuzick model thereafter [19, 20]. Data was censored on 30/04/19.

Updated BC risk information was obtained from a sub-set of FHRPC attendees enroled in the Family History Risk (FHRisk) Study in 2010–11 as described previously [21, 22]. Women were eligible for the FHRisk Study if they were 20-79 years of age and had not previously had BC. This questionnaire (Supplementary Fig. 2) included questions on self-reported weight at date of completion and age 20 years, and current average weekly units of alcohol.

### Data cleaning

Alcohol units for the FHRPC entry questionnaire were calculated as pint of lager = 3 units, 175 ml glass wine = 2 units, glass of spirits = 1 unit as per guidance given on the FHRisk questionnaire. The following data points were excluded: alcohol units >100 units/week (*n* = 1 at FHRPC entry and *n* = 1 at FHRisk), cigarettes smoked >100 per day (*n* = 1 at FHRPC entry, *n* = 0 at FHRisk). BMI was categorised as per World Health Organisation criteria (underweight <18.5 kg/m^2^, healthy weight 18.5–24.9 kg/m^2^, overweight 25–29.9 kg/m^2^, obese ≥30.0 kg/m^2^), and alcohol consumption as per National Institute of Health and Care Excellence (NICE) criteria (non-drinker, low risk ≤14 units/wk, hazardous 14.1–35 units/wk, harmful ≥35 units/wk) [23, 24]. Deprivation measure was English Indices of Multiple Deprivation (IMD) 2015 rank identified from participant postcodes via Geoconvert [25]. Ethnicity data were categorised as per the 2021 Census of England and Wales [26]. Weight change data were included in the analyses if there was >12 months between the two time points, i.e., between FHRPC entry and the FHRisk study in 2010–11, age 20 and FHRPC entry, and age 20 and the FHRisk study.

### Statistics

Normally distributed data are presented as mean and standard deviation (SD), otherwise median and interquartile range (IQR, 25th and 75th percentiles) are presented. Categorical data are presented as number and percentage.

#### 1) Weight, BMI and health behaviours amongst women joining the FHRPC, 1989-2018

We assessed weight and health behaviours in women joining the clinic in entry periods 1989–1998, 1999–2008, 2009–2018. This excluded *n* = 25 eligible for analysis that joined in 1987/88 and *n* = 47 that joined in 2019 before censoring (total *n* = 72).

Trends in BMI, alcohol and smoking for women joining the clinic between 1989 and 2018 were characterised with age-period-cohort analysis using the Partial Least Squares (PLS) approach as described in Jiang et al. [27]. Any observed differences in weight or behaviours over time are likely to be complex. PLS allows estimation of the separate effects of age (age at FHRPC entry), the year of FHRPC entry (period) and birth year (cohort), and to accommodate curvilinear effects. Separating out these effects will aid identification of at-risk groups that could be targeted for health interventions. Age-related effects refer to individual-level changes in weight, alcohol and smoking trajectories throughout adulthood. Period-related effects refer to external influences on these three outcomes measures, for example social, economic, cultural and physical environments. For example, the effects of the recent obesogenic environment on weight gain, and effects of legislation, taxation and societal norms on smoking and alcohol behaviours. Cohort effects could be due to differences in exposure to, for example, childhood physical activity opportunities [28]. For each of the three outcome measures (weight, smoking and alcohol) PLS is used to obtain estimates for age (age at FHRPC entry, 20–60 years), period (year of FHRPC entry, 1990–2018 [removed years with small numbers of data points: first three years of clinic opening 1987–1989, and 2019 which was incomplete due to censor]) and cohort (birth year). We began with linear PLS analysis for BMI by including age at FHRPC entry, year of FHRPC entry and birth year as covariates. Since PLS penalises against variables with comparatively smaller variances, all predictor variables were scaled prior to running PLS. The number of components extracted for the PLS was based on finding the dimension with the lowest cross validation error. Dummy variables were created for all the predictor variables (one for each year) to explore potential curvilinear effects. No dummy variables were created for age 20 at joining FHRPC, FHRPC joining year (period) 1990 and birth year (cohort) 1930 for reasons of identifiability; no other constraints were placed on the dummy variables, since this is not required for PLS. Having obtained coefficients for the dummy variables, Locally Weighted Scatterplot Smoothers (LOESS) were applied to identify curvilinear effects. The resulting fitted curves were overlaid on the scatter plot of the parameter estimates to allow for visual comparisons. Participants with missing values in BMI, alcohol or smoking are excluded from the analyses.

#### 2) Changes in weight and BMI: (a) after joining the FHRPC, and (b) during adulthood from age 20

Characteristics of the FHRisk population were compared to the rest of the eligible FHRPC population using independent samples t-tests and Mann-Whitney U Tests for parametric and non-parametric continuous data respectively, and Pearson Chi-Squared tests for categorical data. Data from all three time points (age 20, FHRPC entry, FHRisk completion) was tabulated for all women returning a valid FHRisk questionnaire to highlight changes over time in this population. Women with valid self-reported BMI for at least two of the timepoints of interest were included in cross tabulation of BMI categories (*n* = 2243 FHRPC entry to FHRisk, *n* = 2407 age 20 to FHRPC entry, *n* = 2883 age 20 to FHRisk). Percentage weight change and weight change per year were calculated using all available data between each of the three time points. ANCOVAs were used to assess whether weight changes between the three timepoints had changed from the earliest to the latest joining periods, using the 2009–2018 entry period as the reference category and including the covariates weight at first time point, duration between the two timepoints, and IMD decile. For the ANCOVAs, missing data was imputed by predictive mean matching for continuous variables, and logistic regression for the binary variable ‘smoking history’. The data was assumed missing at random, and a predictor matrix was specified a priori. Fifteen imputed datasets were created, and models fit to each separately, before pooling estimates using Rubin’s rules. Parameter estimates with associated 95% confidence intervals are presented. We also tabulated data from women with complete BMI data for all three time points to show changes in their BMI categories (*n* = 2075). McNemar-Bowker tests used for pairwise comparisons of the BMI categories at the three timepoints.

#### 3) Changes in alcohol consumption after joining the FHRPC

Alcohol units per week and adherence to alcohol recommendations at FHRPC entry and FHRisk completion were tabulated for all women returning a valid FHRisk questionnaire to highlight changes over time in this population.

Analysis was performed using SPSS 25 (IBM, New York, USA) apart from PLS regression and graphics which were performed using the PLS library in the statistical package, R, and the multiple imputation methods which used the *mice* package in R (version 4.1.0, https://www.r-project.org/index html).

## Results

### 1) Weight, BMI and health behaviours amongst women joining the FHRPC, 1989-2018

**Fig. 1:**
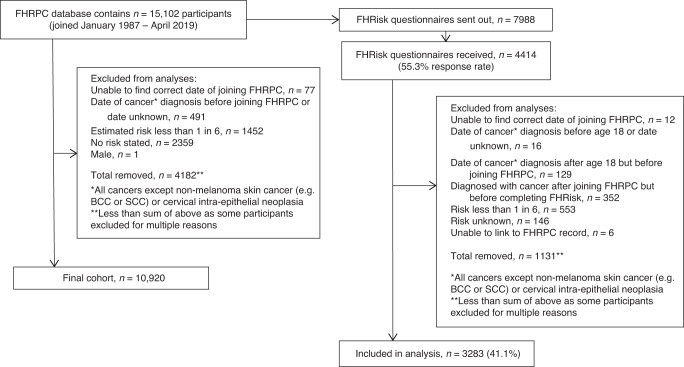
Flow diagram for FHRPC and FHRisk analysis combined.

At date of censor (30/04/19), the FHRPC database contained 15,102 subjects of which 10,920 were eligible for inclusion in the analyses (Fig. 1). Average age at FHRPC entry was consistent across the periods and 35.9% of women in the most recent period were ≤35 years (Table 1). Median BC risk was 33.3% (IQR 20.0–33.3) meaning that women had a median 1 in 3 lifetime chance of developing BC. The proportion of women from the two most deprived quintiles increased over time as did the proportion from non-white ethnicity groups.

**Table 1:** Baseline characteristics for full valid FHRPC population and by period of FHRPC entry.

	Full population	1989–1998	1999–2008	2009–2018
Number of participants	10,920*	3041	3443	4364
Age at FHRPC entry^1^	39.6 (9.2)	39.2 (8.9)	39.2 (9.0)	40.2 (9.4)
Missing	*n* = 0			
Age ≤35 years^2^	4040 (37.0%)	1174 (38.6%)	1276 (37.1%)	1566 (35.9%)
Estimated lifetime BC risk (%)^3,4^	33.3 (20.0–33.3)	25.0 (16.7-33.3)	33.3 (25.0–33.3)	30.0 (23.3-33.3)
IMD quintile^2^				
1 (most deprived)	2125 (20.5%)	483 (17.6%)	675 (20.4%)	953 (22.6%)
2	1856 (17.9%)	447 (16.3%)	602 (18.2%)	797 (18.9%)
3	1772 (17.1%)	473 (17.2%)	585 (17.7%)	703 (16.6%)
4	2101 (20.3%)	622 (22.6%)	625 (18.9%)	841 (19.9%)
5 (least deprived)	2490 (24.1%)	722 (26.3%)	931 (22.0%)	931 (22.0%)
Missing	*n* = 576	*n* = 294	*n* = 141	*n* = 139
IMD rank (lower = more deprived)^3^	17,754 (8133–26,077)	19,304 (9812–26621) *n* = 294	17,674 (8129–26,227) *n* = 141	16,441 (7529–25,675) *n* = 139
Missing	*n* = 576			
Ethnicity				
White	6443 (94.1)	2388 (98.5)	2290 (95.3)	1732 (87.4)
Asian or Asian British	173 (2.5)	15 (0.6)	38 (1.6)	119 (6.0)
Black, Black British, Caribbean or	61 (0.9)	7 (0.3)	21 (0.9)	33 (1.7)
African				
Mixed or multiple ethnic groups	75 (1.1)	8 (0.3)	18 (0.7)	48 (2.4)
Other ethnic group	92 (1.3)	6 (0.2)	36 (1.5)	50 (2.5)
Missing	*n* = 4076	*n* = 617	*n* = 1040	*n* = 2382
Height (m)^1^	1.64 (0.07)	1.63 (0.07)	1.64 (0.07)	1.65 (0.07)
Missing	*n* = 2596	*n* = 190	*n* = 669	*n* = 1706
Weight (kg)^3^	64.4 (57.6-74.4)	64.8 (57.2–69.9)	68.4 (58.1–75.3)	70.7 (59.0–78.9)
Missing	*n* = 3656	*n* = 414	*n* = 1281	*n* = 1923
BMI (kg/m^2^)^3^	24.0 (21.6–27.6)	23.4 (21.5–26.3)	24.3 (21.9–27.9)	24.7 (22.0–28.9)
Missing	*n* = 3688	*n* = 420	*n* = 1290	*n* = 1939
BMI categories^2^:				
Underweight (<18.5 kg/m^2^)	165 (2.3%)	59 (2.3%)	39 (1.8%)	66 (2.7%)
Healthy weight (18.5–24.9 kg/m^2^)	4080 (56.4%)	1664 (63.5%)	1181 (54.9%)	1214 (50.1%)
Overweight (25–29.9 kg/m^2^)	1851 (25.6%)	621 (23.7%)	577 (26.8%)	647 (26.7%)
Obese (≥30.0 kg/m^2^)	1136 (15.7%)	277 (10.6%)	356 (16.5%)	498 (20.5%)
Missing	*n* = 3688	*n* = 420	*n* = 1290	*n* = 1939
Alcohol categories^2^:				
Non-drinker	3034 (37.7%)	864 (30.7%)	1113 (42.8%)	1039 (40.2%)
Low risk (≤14 units/wk)	3572 (44.4%)	1384 (49.2%)	984 (37.8%)	1187 (45.9%)
Hazardous (14.1–35 units/wk)	1249 (15.5%)	501 (17.8%)	431 (16.6%)	313 (12.1%)
Harmful (≥35 units/wk)	184 (2.3%)	66 (2.3%)	73 (2.8%)	45 (1.7%)
Missing	*n* = 2881	*n* = 226	*n* = 842	*n* = 1780
Alcohol units per week (excluding categories of non-drinkers and missing)^3^	9.0 (4.0–16.0)	9.0 (5.0–16.0)	10.0 (6.0–18.0)	8.0 (4.0–14.0)
Smoking status^2^:				
Non-smoker	3611 (53.0%)	1405 (52.4%)	997 (51.0%)	1190 (55.6%)
Former smoker	1470 (21.6%)	379 (14.1%)	478 (24.4%)	607 (28.4%)
Current smoker	1732 (25.4%)	899 (33.5%)	481 (24.6%)	344 (16.1%)
Missing	*n* = 4107	*n* = 358	*n* = 1487	*n* = 2223
Pack-years (former and current smokers only)^3^	7.5 (2.5–15.0)	9.8 (3.5–18.0)	7.5 (2.5–15.0)	5.2 (2.3–12.4)
Missing	*n* = 69	*n* = 27	*n* = 18	*n* = 24

^1^Mean (SD), ^2^n (%), ^3^median (IQR: 25th and 75th percentiles), ^4^based on a modification of the Claus tables until 2003 and the Tyrer-Cuzick model thereafter.

^*^Total is greater than the sum of joining periods as *n* = 25 women joined 1987–88, and *n* = 47 joined in the first part of 2019.

Height, weight and BMI are higher in the more recent entry periods (Table 1). The prevalence of obesity has nearly doubled with an increase from 10.6% in the 1989–98 period to 20.5% in the 2009–18 period. Alcohol intake above the UK recommended maximum of 14 units per week reduced from 20.1% in the first period to 13.8% in the most recent period, and current smoking from 33.5% to 16.1%.

**Fig. 2:**
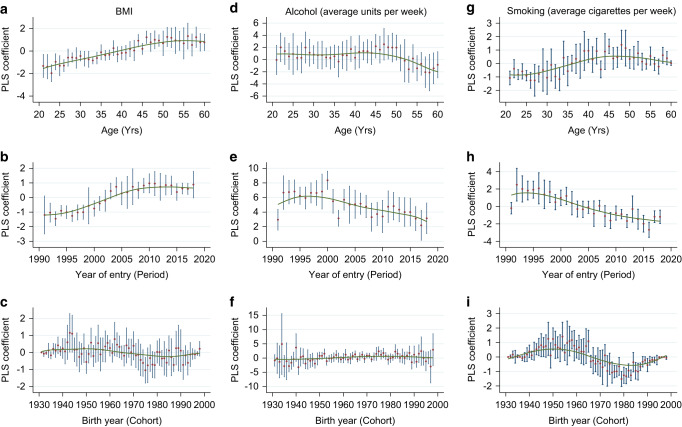
Partial least squares analyses. LOESS curves for age, period and cohort effects for BMI at FHRPC entry (**a**–**c**), alcohol intake at FHRPC entry (**d**–**f**) and weekly cigarette smoking at FHRPC (**g**–**i**).

LOESS curves for BMI at FHRPC entry (Fig. 2a–c) show the increase in BMI with age of FHRPC entry up to around age 53 after which it levels off and shows a slight decrease in older women (Fig. 2a), and an increase over time with some levelling off and a slight decrease from around 2010 (Fig. 2b). There was no association with birth year (cohort, Fig. 2c). Linear analysis in Table 2 revealed a statistically significant relationship for all three variables. Women who were older when they joined the FHRPC had a higher BMI than younger women (0.027 BMI units/y, 95% CI 0.017–0.037), BMI increased by 0.049 units for each year of entry (95% CI 0.039–0.059), and there was a higher BMI in women who were born later (0.022 BMI units/y, 95% CI 0.014–0.031).

**Table 2:** Output from linear partial least squares analysis for age, period and cohort effects.

Variables	BMI (kg/m^2^ per year) Coefficient (95%CI)	Alcohol (average weekly units per year) Coefficient (95%CI)	Smoking (average weekly cigarettes per year) Coefficient (95%CI)
Age	0.027 (0.017 to 0.037)	–0.459 (–0.576 to –0.343)	0.623 (0.476 to 0.783)
Year of FHRPC entry (Period)	0.049 (0.039 to 0.059)	–0.751 (–0.867 to –0.635)	–0.783 (–0.873 to –0.693)
Birth year (Cohort)	0.022 (0.014 to 0.031)	–0.191 (–0.258 to –0.123)	–1.021 (–1.165 to –0.875)

Alcohol intake at entry is associated with age and year of entry (Fig. 2d–f). Intake remains steady until around age 45 then decreases with an overall decrease of 0.459 (95% CI –0.576 to –0.343) weekly units per year of age. There was an increase in alcohol intake to around 1996 then a steady decrease, with an overall decrease of 0.751 (95% CI –0.867 to –0.635) weekly units per year over the duration of the analysis. Linear analysis also showed a decrease in alcohol consumption in women with a later birth year (–0.191, 95% CI –0.258 to –0.123).

Weekly cigarette smoking at entry is also associated with all three variables of age, year of entry (period), and birth year (cohort) (Fig. 2g–i). Number of weekly cigarettes smoked increased to around age 45 then decreased with an overall increase over the study duration of 0.623 (95% CI 0.476–0.783) weekly cigarettes per year of age. There are decreases in weekly cigarettes smoked with both more recent year of entry (–0.783/year, 95% CI –0.873 to –0.693), and more recent birth year (–1.021/year, 95% CI –1.165 to –0.875).

### 2a) Changes in weight and BMI after joining the FHRPC

There was a 55.3% response rate to the FHRisk questionnaire with *n* = 3283 valid responses (Fig. 1). Compared with the rest of the eligible FHRPC population, FHRisk responders were marginally older and had higher BC risk (Supplementary Table 2). They were also more affluent, more likely to be of white ethnicity, had a lower median BMI at FHRPC entry and a lower proportion had obesity, were more likely to be a non-drinker but had a higher weekly alcohol intake amongst those who consumed alcohol.

After FHRPC entry, women’s weight and BMI increased (Table 3). Amongst women returning a valid FHRisk questionnaire, median weight at FHRPC entry (mean age 40.2 ± 8.7 years) was 63.5 (IQR 57.2-72.6) kg and 38.1% had overweight or obesity, which had increased to 67.6 (60.3-77.6) kg and 51.8% by mean age 51.0 (±10.5) years.

**Table 3:** Self-reported characteristics and health behaviours of women at age 20, FHRPC entry and completion of FHRisk questionnaires (*n* = 3283 women returning a valid FHRisk questionnaire).

	Timepoint		
	Age 20	FHRPC Entry	FHRisk
Age^1^	–	40.2 (8.7)	51.0 (10.5)
Time since FHRPC entry (months)^3^	–	–	126 (66–191)
Weight (kg)^3^	57.2 (52.6–63.5)	63.5 (57.2–72.6)	67.6 (60.3–77.6)
	Missing	Missing	Missing
	*n* = 355	*n* = 935	*n* = 152
BMI (kg/m^2^)^3^	21.6 (20.2–23.5)	23.7 (21.6–26.9)	25.2 (22.6–28.9)
	Missing *n* = 361	Missing *n* = 937	Missing *n* = 159
BMI categories^2^:			
Underweight (<18.5 kg/m^2^)	214 (7.3)	48 (2.0)	39 (1.2)
Healthy weight (18.5–24.9 kg/m^2^)	2294 (78.5)	1404 (59.8)	1466 (46.9)
Overweight (25–29.9 kg/m^2^)	315 (10.8)	594 (25.3)	979 (31.3)
Obese (≥30.0 kg/m^2^)	99 (3.4)	300 (12.8)	640 (20.5)
Alcohol units per week^3^	–	3.0 (0.0–11.0)	4.0 (1.0–10.0)
		Missing *n* = 295	Missing *n* = 135
Alcohol categories^2^:	–		
non-drinker		1269 (42.5)	775 (24.6)
sensible (≤14 units/wk)		1188 (39.8)	1919 (61.0)
hazardous (14.1–35 units/wk)		465 (15.6)	426 (13.5)
harmful (≥35 units/wk)		66 (2.2)	28 (0.9)
		Missing *n* = 295	Missing *n* = 135

^1^Mean (SD), ^2^n (%), ^3^median (IQR: 25th and 75th percentiles).

Twenty-eight percent of women who were a healthy BMI at FHRPC entry had increased to overweight or obese by FHRisk completion, and 30% of those with overweight at FHRPC entry had developed obesity by FHRisk completion. However, less than 12% of those with overweight or obesity at FHRPC entry had shifted into a lower category by FHRisk completion (Supplementary Table 3a). Women gained 4.5 (IQR 0.0–12.5) % weight between FHRPC entry and FHRisk completion and weight gain per year was 0.25 (0.00–0.68) kg (Supplementary Table 4).

Analysis using ANCOVAs with multiple imputation (Table 4, with complete case analysis in Supplementary Table 5) revealed no significant differences in weight change after FHRPC entry between the three joining periods, after adjusting for time between Clinic entry and FHRisk completion.

**Table 4:** Comparison of weight changes over the three joining periods: ANCOVA (multiple imputation).

			Parameter estimates					Estimated marginal means		
	*n*	period	B (Regression Coefficient)	Std Error	Sig.	95% Confidence Interval		Mean Change (kg)	95% Confidence Interval	
						Lower Bound	Upper Bound		Lower Bound	Upper Bound
Weight change FHRPC entry to completing FHRisk questionnnaire^a^	1393	1989–1998	1.7	1.1	0.15	–0.6	4	4.1	3.3	5.0
	1490	1999–2008	0.7	0.7	0.31	–0.6	2	3.2	2.6	3.7
	391	2009–2018	Ref					2.5	0.9	4.0
Weight change age 20 to FHRPC entry^b^	3041	1989–1998	–2.7	0.3	<0.001	–3.3	-2	6.4	5.9	6.8
	3443	1999–2008	–0.6	0.3	0.06	–1.1	0	8.5	8.0	8.9
	4364	2009–2018	Ref					9.0	8.5	9.6
Weight change age 20 to completing FHRisk questionnaire^c^	1393	1989–1998	0.3	0.7	0.70	–1.2	1.8	11.1	10.4	11.7
	1490	1999–2008	0.4	0.7	0.54	–0.9	1.7	11.2	10.6	11.8
	391	2009–2018	Ref					10.8	9.5	12.0

^a^Weight change FHRPC entry to FHRisk completion (kg): Dependent variable: weight change FHRPC entry to FHRisk completion / Fixed factor: period group / Covariates: weight at FHRPC entry, duration between FHRPC entry and FHRisk completion, IMD decile.

^b^Weight change age 20 to FHRPC entry (kg): Dependent variable: weight change age 20 to FHRPC entry / Fixed factor: period group / Covariates: weight age 20, duration between age 20 and FHRPC entry, IMD decile.

^c^Weight change age 20 to FHRisk completion: Dependent variable: weight change age 20 to FHRisk completion / Fixed factor: period group / Covariates: weight at age 20, duration between age 20 and FHRisk completion, IMD decile.

### 2b) Changes in weight and BMI during adulthood from age 20

Over 30% of women with a healthy BMI at age 20 had shifted into the overweight or obese categories by the time of FHRPC entry (Supplementary Table 3b). Only 18% of women who were overweight at age 20 had shifted down into the healthy weight category by the time of FHRPC entry. The uplift in BMI category between age 20 and FHRisk completion was even more pronounced. Forty-seven percent of women with a healthy BMI at age 20 had developed overweight or obesity by the time of the FHRisk study, and 58% of those with overweight had developed obesity (Supplementary Table 3c). Women gained median 9.7 (IQR 1.4–20.6) % weight during the 20.1 (±8.5) years from age 20 and FHRPC entry, or 0.28 (0.04–0.60) kg per year (Supplementary Table 4). This had increased to 15.8 (IQR 6.2–28.7) % weight by the time of FHRisk. Analysis using ANCOVAs with multiple imputation (Table 4, with complete case analysis in Supplementary Table 5) adjusted for duration between timepoints revealed that the most recent joining period had gained more weight between age 20 and joining the FHRPC than the previous two periods (9.5 [8.3–10.6] kg for 2009–2018 period vs. 8.0 [7.4–8.7] kg [*p* = 0.03] and 6.0 [5.5–6.6] kg [*p* < 0.001] for 1999–2008 and 1989–1998 periods respectively). There were no significant differences in patterns of weight change between the three FHRPC entry periods between the other time points: age 20 or FHRPC entry to FHRisk completion.

Data from participants with BMI at all three time points (*n* = 2075, Supplementary Table 6) shows that prevalence of obesity trebled between age 20 and FHRPC entry (mean age 39.6 [SD 8.8], 3.6% to 12.1%) and increased again to FHRisk completion (mean age 51.4 [SD 10.4], 20.2%).

### 3) Changes in alcohol consumption after joining the FHRPC

Between FHRPC entry and completion of FHRisk there was a small increase in median weekly alcohol. The proportion of non-drinkers reduced from 42.5% to 24.6% and the number of women reporting drinking within recommended levels increased from 39.8% to 61.0% (Table 3). The proportion of women drinking at hazardous or harmful levels (>14 units per week) also reduced between joining FHRPC and completion of FHRisk, however, 14.4% of FHRisk responders still consumed alcohol above the recommended maximum level.

## Discussion

This observational study has reported that the prevalence of smoking and alcohol intake above recommended levels have both decreased amongst women entering the Manchester Family History, Risk and Prevention Clinic since its launch in 1987. However there has been a significant increase in the prevalence of overweight and obesity on joining the clinic.

Our PLS analysis showed the increase in BMI at FHRPC entry levels off from around 2010 which is also indicated by Health Survey for England (HSE) figures for women in our FHRPC joining age group (35–44 and 45–54 year old women) [29]. Unlike in the HSE which then shows a further increase in BMI from 2015, no further increase in BMI at FHRPC entry after 2010 is indicated by the PLS analysis. The increase in BMI to around age 53 is consistent with data from the HSE which shows the highest mean BMI in women is in 45–54 year olds, with a decline in older age groups [29].

The increase in weight and BMI in women at increased risk of BC upon joining the FHRPC is in line with increases seen amongst women in the general population in England, though the actual levels seen in the FHRPC are lower. Our data show that obesity levels at FHRPC entry amongst women with a mean age of 40 years have risen from 11% in our 1989–1998 joining period to 21% in our 2009–2018 period, compared to 35–44 year old UK women in the HSE which shows an increase from 17% in 1993 to 25% in 2014 (the middle years of our joining periods) [29]. Our lower levels of obesity could be due to the FHRPC including fewer women from more deprived areas. The reasons for this weight gain over time are complex and interlinked, as detailed in the obesity system map of the Foresight Report [30]. This ranges from aspects of social psychology, for example exposure to food advertising and peer pressure, to levels of satiety, resting metabolic rate and other physiological aspects. The gains in weight we have observed have implications for public health due to the increased risks of links between many forms of ill health and overweight and obesity. The current annual cost to the NHS of dealing with overweight and obesity-related conditions is estimated to be over £6 billion [31].

The decrease in smoking incidence over time amongst women at clinic entry fits with national trends of a fall in smoking rates. The proportion of current smoking behaviour in our most recent period is 16% compared to 19% in 35–44 year-old women in England in 2013 (the middle year of our most recent period) [29]. Our findings align with those of previous studies of women at increased risk of BC which have also found lower rates of smoking compared to the general population [32, 33]. This may be associated with lower proportion of women from more deprived areas in high-risk clinics or because many women at increased risk of BC perceive smoking as a more important risk factor for developing BC than weight [32, 33].

Women in our FHRPC report lower levels of alcohol consumption compared with those reported by women in the general population. Fourteen percent of women in the most recent FHRPC entry period (2009–2018) consumed alcohol above recommended limits at FHRPC entry compared with 17% of 35–44 year-old women in England in 2013 [29]. Respectively, 40% and 19% of these periods reported no alcohol intake [29]. Our PLS analysis showed a reduction in alcohol intakes at FHRPC entry from around 1996 and age 45, which are also not mirrored by HSE data which shows an increase in women between 1993 and 2002 [34] and the highest mean units of alcohol in women aged 55-64 years [29]. The lower level of alcohol consumption amongst women in our FHRPC may be a result of the small amount of education on health behaviours that women receive in the form of standard leaflets, and in some cases a discussion, at FHRPC entry. This may raise awareness above levels seen in women attending genetics clinics, breast screening and symptomatic clinics where it has been shown that alcohol is not generally recognised as a BC risk factor [33, 35, 36]. It may be due to reporting bias, or a difference in methodology between the two datasets. Alcohol intake at FHRPC entry was collected as self-reported average drinks per week (pints of beer/lager, glasses of wine, glasses of spirits) compared with the more detailed face-to-face interviews of the HSE. The reasons for the apparent reduction in alcohol consumption in our more recent FHRPC entry periods are unclear but welcomed in the light of the increased risk of breast and other cancers with any alcohol intake [37].

Our analyses have highlighted the issue of adult weight gain in women at increased risk of BC both in the time between young adulthood (age 20 years) and joining the Clinic (mean 40.2 [SD 8.7] years), and also after joining (to mean 51.0 [10.5] years). We have shown that the increase in weight from age 20 to FHRPC entry is higher now than in earlier entry periods. The annual self-reported weight gain in the present population between FHRPC entry (mean 40.2 [SD 8.7] years) and FHRisk completion (mean 51.0 [SD 10.5] years) was 0.25 (IQR 0.00–0.68) kg/yr which is slightly less than that for women of comparable age in the EPIC-Oxford longitudinal study (mean 0.45 kg/yr, 95% CI 0.42–0.49, *n* = 16,593). The majority of adult weight gain occurred before presentation at the FHRPC. We have previously highlighted the time between ages 18 and 35 as the predominant time for weight gain in a woman’s lifespan, and the complexity of reasons for this [38].

These analyses show that many women shifted from not consuming alcohol upon joining the FHRPC, to drinking at ‘sensible’ levels at the time of the FHRisk study. This has implications for cancer risk amongst this population which increases even at recommended levels [39]. In addition, high alcohol intakes persist amongst a minority of women in the FHRPC with 14.4% consuming alcohol above recommended levels.

### Strengths

The strengths of the current analysis are the unique long-term, longitudinal data from high-risk women without cancer from a large UK FHRPC. We include data on deprivation and ethnicity and highlights issues that are likely to be relevant to other UK FHRPCs. Updated data after having been in the FHRPC for median 10 years was available on over 3000 patients.

### Limitations

There are a number of limitations, firstly regarding the study design and analyses. The observed BMI and behaviours are reported in the clinic attendees. We did not include a population control to see whether trends are specific to the FHRPC population or observations in the general population. The PLS graphs separate out the effects of age, year of FHRPC entry (period) and birth year (cohort) but are not adjusted for other confounders such as deprivation or smoking. This is an exploratory approach reflecting a non-linear trend which we applied only to cases with complete data. In our final analysis we were only able to use *n* = 7009, 7779 and 2971 values of BMI, alcohol and smoking respectively.

Secondly, there were issues with data collection. Weight and health behaviours were all self-reported which may affect the validity of the current findings. Previous studies suggest that women with overweight and obesity are more likely to underestimate their current weight [40]. However a metanalysis has shown that recalled weights from early adulthood to be fairly accurate [41]. Self-reported alcohol is considered fairly accurate [42], but potentially less accurate amongst high drinkers [43]. Wording of the alcohol questions differed between the FHRPC entry and FHRisk questionnaires which may affect the two datasets. The initial FHRPC entry questionnaire asked participants to enter number of glasses of wine and spirits but unlike the FHRisk questionnaire it did not provide guidance on size of glass. Increased glass sizes over time may have introduced errors in this part of the analysis [44, 45].

Thirdly there are limitations regarding missing data. Response to the FHRisk questionnaire was 55.3% and this will affect the validity of the results. With fewer women completing, the precision of the estimates will be reduced, and it is unknown how the accuracy will be affected. There were differences between responders and non-responders with responders as detailed in results section 2a and this may bias the results. It is possible that the analyses and interpretations from the updated information are not generalisable to all clinic attendees. We accept that missing data can substantially reduce the sample size and may cause bias and a reduction in efficiency. Unbiased results can be obtained with large proportions of missing data provided the imputation model is properly specified and data is missing at random. Using the imputation model, the missing data are filled in to generate complete data sets. The complete data sets are then analysed with standard procedures using the analysis model and results from the complete data sets are combined for inference using Robin’s rules.

However, in our data the setup is not as straightforward. In order to detect trends in the data, we fitted models using Partial Least Squares to age at entry, year of entry and birth year using dummy variables for all three. This introduces a large number of parameters, but using a principal components type of approach will reduce the dimensionality of the problem and hence the number of components needed to estimate the model. Cross Validation guided us to the best number of components to use, then we fitted the model and used jacknifing/simulation to get the standard errors.

Even if we had generated multiple copies of the data using imputation, repeating all these steps separately on each imputed data set is not the same as doing it simultaneously in an all-encompassing analysis model, which we readily accept as a limitation of the paper. There was just not a simple approach that could be used to address this given the complexity of the model that was fitted to the data.

We have not reported on physical activity behaviours as this data was not collected at clinic entry. Likewise, we have not reported changes in smoking behaviour after joining the clinic as smoking data not collected as part of the FHRisk study. Our analyses and interpretations of the data could have been affected by a lack of information on comorbidities, medications and other confounders that could affect weight, such as level of education.

Lastly there are issues around the generalisability of the current findings. Thirty-seven percent of ethnicity data was missing. Amongst those providing ethnicity data, 94% were white, compared with 82% of the population of England and Wales according to the 2021 census [46]. This reflects the wider issue of underrepresentation of ethnic minority groups engaging with health research and attending high risk BC clinics and breast screening [47, 48]. We have reported a positive change over the past three decades with a higher representation from minority ethnic groups in the most recent joining period. A further impact on generalisability is that fewer FHRisk responders were from the most deprived quintile.

Weight gain and unhealthy behaviours are an issue amongst women attending this FHRPC as shown by the increase in BMI and the decrease in proportion of women not drinking alcohol after joining. FHRPCs offer the opportunity to engage their patients with weight control and healthy behaviours such as achieving physical activity recommendations and limiting smoking and alcohol, which could reduce the future burden of BC, other cancers and other diseases such as diabetes and cardiovascular disease. UK familial BC guidelines recommend that women should be advised on the increased BC risk associated with overweight/obesity, sedentary lifestyle, smoking and alcohol [12]. Current standard care involves general written advice which is likely to have a minimal effect on health behaviours [49]. Thus, current approaches are unlikely to adequately manage risk in the FHRPCs and this opportunity is being missed.

### Implications and future research

Women at increased risk of BC should have access to evidence-based interventions to support them with healthy behaviours, starting in early adulthood to reduce weight gain and lifetime exposure to behavioural risk factors and excess weight, and continuing through adulthood. Future research could build on the work of our group [50] and others [51–53] to find cost-effective interventions for tackling existing weight issues or for the primary prevention of weight gain that could be delivered across UK FHRPCs.

Approximately a third of women joining the FHRPC are ≤35 years and in light of the evidence suggesting that this time is important for weight gain, these women should be specifically targeted with evidence-based interventions promoting healthy behaviours and weight management to help these young women avoid the common problem of weight gain. FHRPCs should attempt to engage more women at younger ages so that this support can be offered to all those who would benefit. Our previous interview study reported that young women (aged 25–35 years) at increased risk of BC are interested in joining a program to prevent weight gain and promote healthy behaviours which could be accessed remotely, potentially via an app [54]. A search of existing literature and apps currently available found that there was nothing evidence-based and grounded in psychological theory that would be suitable for testing within this population [55].

The demographics of our population at clinic entry showed that there have been positive changes in the proportions of women attending who have postcodes within the most deprived areas, and who are of non-white ethnicity. However, more improvements could be made to further reduce health inequalities in the UK.

## Supplementary information

Supplementary File List

Supplementary tables and figures

## Data Availability

Data will be made available upon reasonable request.
