# Supplementary material for: Weight, weight gain and behavioural risk factors in women attending a breast cancer family history, risk and prevention clinic: an observational study
Source: BJC Rep. 2024 Mar 14;2:22. doi: 10.1038/s44276-024-00039-9 (PMC11523958; doi:10.1038/s44276-024-00039-9)
Supplement: Supplementary file 1 — Supplementary File List [file 44276_2024_39_MOESM1_ESM.docx]

**Supplementary File List**

Supplementary Figure 1: Relevant excerpts from the FHRPC entry questionnaire

Supplementary Figure 2: FHRisk Questionnaire

Supplementary Table 1: STROBE Statement for cohort studies

Supplementary Table 2: Comparison of eligible FHRisk responders with other eligible FHRPC patients

Supplementary Table 3: BMI category transitions between time points

Supplementary Table 4: Weight change percentage, and weight change per year

Supplementary Table 5: Comparison of weight changes over the three joining periods: ANCOVA (using all available data)

Supplementary Table 6: BMI at each point for those with no missing BMI data (n=2,075), n (%)
