# Supplementary material for: Weight, weight gain and behavioural risk factors in women attending a breast cancer family history, risk and prevention clinic: an observational study
Source: BJC Rep. 2024 Mar 14;2:22. doi: 10.1038/s44276-024-00039-9 (PMC11523958; doi:10.1038/s44276-024-00039-9)
Supplement: Supplementary file 2 — Supplementary tables and figures [file 44276_2024_39_MOESM2_ESM.docx]

**Figures and Tables**

**Supplementary Table 1: STROBE Statement for cohort studies**

|  | Item No | Recommendation | Page No |
| --- | --- | --- | --- |
| **Title and abstract** | 1 | (*a*) Indicate the study’s design with a commonly used term in the title or the abstract | 1 |
|  |  | (*b*) Provide in the abstract an informative and balanced summary of what was done and what was found | 2 |
| Introduction | | | |
| Background/rationale | 2 | Explain the scientific background and rationale for the investigation being reported | 2 |
| Objectives | 3 | State specific objectives, including any prespecified hypotheses | 3 |
| Methods | | | |
| Study design | 4 | Present key elements of study design early in the paper | 4 |
| Setting | 5 | Describe the setting, locations, and relevant dates, including periods of recruitment, exposure, follow-up, and data collection | 4 |
| Participants | 6 | (*a*) Give the eligibility criteria, and the sources and methods of selection of participants. Describe methods of follow-up | 4 |
|  |  | (*b*) For matched studies, give matching criteria and number of exposed and unexposed |  |
| Variables | 7 | Clearly define all outcomes, exposures, predictors, potential confounders, and effect modifiers. Give diagnostic criteria, if applicable | 4 |
| Data sources/ measurement | 8* | For each variable of interest, give sources of data and details of methods of assessment (measurement). Describe comparability of assessment methods if there is more than one group | 4 |
| Bias | 9 | Describe any efforts to address potential sources of bias | 13 |
| Study size | 10 | Explain how the study size was arrived at | N/A |
| Quantitative variables | 11 | Explain how quantitative variables were handled in the analyses. If applicable, describe which groupings were chosen and why | 5 |
| Statistical methods | 12 | (*a*) Describe all statistical methods, including those used to control for confounding | 5 |
|  |  | (*b*) Describe any methods used to examine subgroups and interactions | 5 |
|  |  | (*c*) Explain how missing data were addressed | 6 |
|  |  | (*d*) If applicable, explain how loss to follow-up was addressed | N/A |
|  |  | (*e*) Describe any sensitivity analyses | N/A |
| Results | | |  |
| Participants | 13 | (a) Report numbers of individuals at each stage of study—eg numbers potentially eligible, examined for eligibility, confirmed eligible, included in the study, completing follow-up, and analysed | Fig 1 |
|  |  | (b) Give reasons for non-participation at each stage | N/A |
|  |  | (c) Consider use of a flow diagram | Fig 1 |
| Descriptive data | 14 | (a) Give characteristics of study participants (eg demographic, clinical, social) and information on exposures and potential confounders | Table 1 |
|  |  | (b) Indicate number of participants with missing data for each variable of interest | Table 1 |
|  |  | (c) Summarise follow-up time (eg, average and total amount) | Table 3 |
| Outcome data | 15 | Report numbers of outcome events or summary measures over time | Table 1 |
| Main results | 16 | (*a*) Give unadjusted estimates and, if applicable, confounder-adjusted estimates and their precision (eg, 95% confidence interval). Make clear which confounders were adjusted for and why they were included | Tables 1, 2, 3, 4  Supplementary tables 2, 3, 4, 5, 6 |
|  |  | (*b*) Report category boundaries when continuous variables were categorized | 4 |
|  |  | (*c*) If relevant, consider translating estimates of relative risk into absolute risk for a meaningful time period | N/A |
| Other analyses | 17 | Report other analyses done—eg analyses of subgroups and interactions, and sensitivity analyses | 5-7 |
| Discussion | | | |
| Key results | 18 | Summarise key results with reference to study objectives | 10-12 |
| Limitations | 19 | Discuss limitations of the study, taking into account sources of potential bias or imprecision. Discuss both direction and magnitude of any potential bias | 12-13 |
| Interpretation | 20 | Give a cautious overall interpretation of results considering objectives, limitations, multiplicity of analyses, results from similar studies, and other relevant evidence | 13 |
| Generalisability | 21 | Discuss the generalisability (external validity) of the study results | 13 |
| Other information | | | |
| Funding | 22 | Give the source of funding and the role of the funders for the present study and, if applicable, for the original study on which the present article is based | 15 |

**Supplementary Figure 1: Relevant excerpts from the FHRPC entry questionnaire**

**
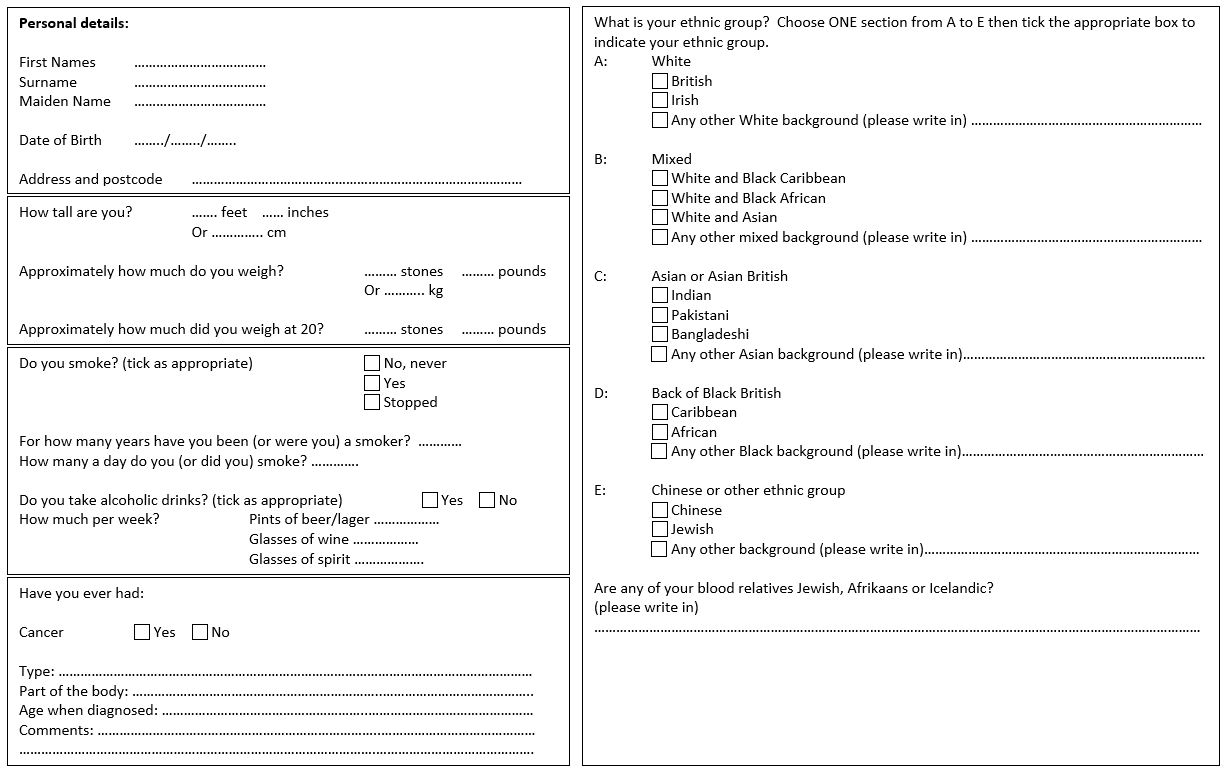
**

**Supplementary Figure 2: FHRisk Questionnaire**


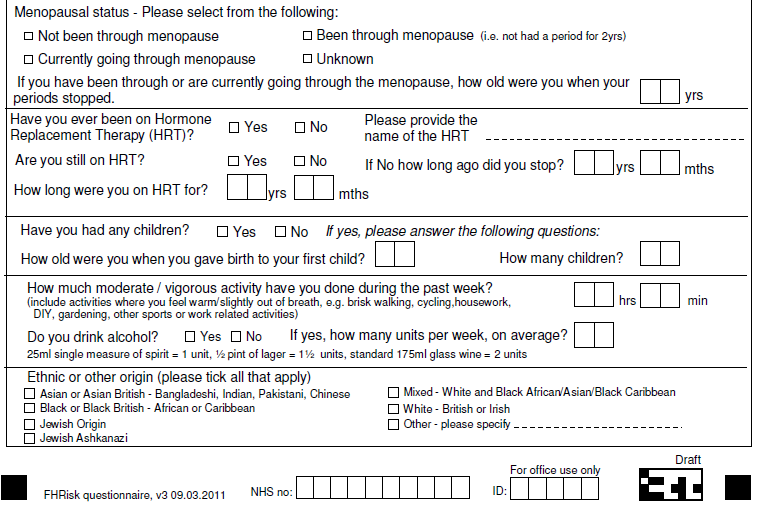

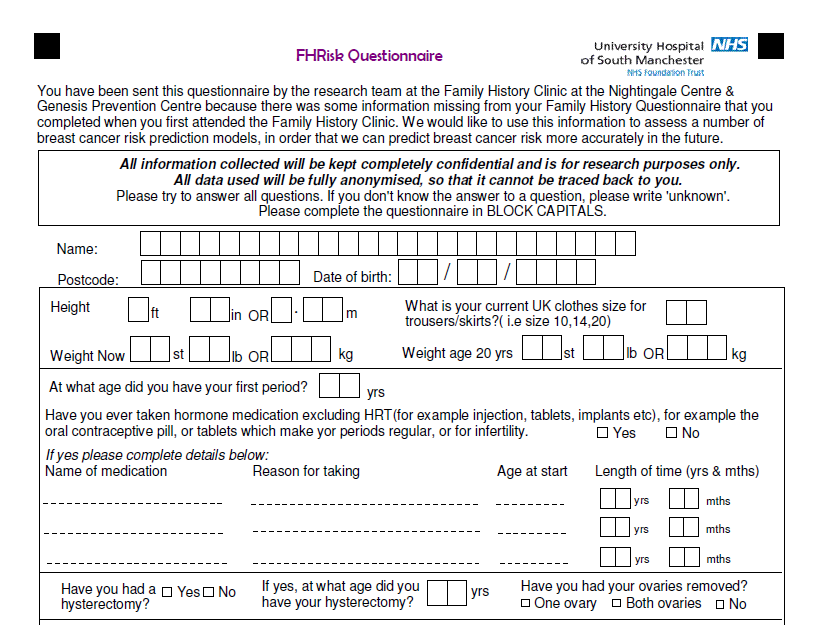


**Supplementary Table 2: Comparison of eligible FHRisk responders with other eligible FHRPC patients**

|  | **FHRisk responders** | **Other eligible FHRPC patients** | **P for difference** |
| --- | --- | --- | --- |
| n | 3283 | 7637 | - |
| Age at FHRPC entry^1^ | 40.2 (8.7) | 39.4 (9.3) | **<0.001** |
| Age ≤35 years at FHRPC entry^2^ | 1073 (37.2%) | 2967 (38.9%) | **<0.001** |
| Age at time of FHRisk survey | 51.0 (10.5) | 43.6 (12.0) | **<0.001** |
| Height (m)^1^  Missing | 1.64 (0.07)  n=30 | 1.64 (0.07)  n=2566 | 0.008 |
| Risk (%)^3^ | 33.3 (20.0-33.3) | 30.0 (20.0-33.3) | 0.004 |
| IMD quintile^2^  1 (most deprived)  2  3  4  5 (least deprived)  Missing | 515 (16.2%)  504 (15.9%)  555 (17.5%)  683 (21.5%)  914 (28.8%)  n=112 | 1610 (22.4%)  1352 (18.8%)  1217 (17.0%)  1418 (19.8%)  1576 (22.0%)  n=464 | **<0.001** |
| Ethnicity  White  Asian or Asian British  Black, Black British, Caribbean or African  Mixed or multiple ethnic groups  Other ethnic group  Missing | 3115 (97.3)  24 (0.7)  14 (0.4)  30 (0.9)  18 (0.6)  n=82 | 3328 (91.4)  149 (4.1)  47 (1.3)  45 (1.2)  74 (2.0)  n=3994 | **<0.001** |
| Weight at FHRPC entry (kg)^3^  Missing | 63.5 (57.2-72.6)  n=935 | 65.3 (58.1-76.2)  n=2721 | **<0.001** |
| BMI at FHRPC entry (kg/m^2^)^3^  Missing | 23.7 (21.6-26.9)  n=937 | 24.1 (21.7-27.9)  n=2751 | **<0.001** |
| BMI categories at FHRPC entry^2^:  Underweight (<18.5 kg/m^2^)  Healthy weight (18.5-24.9 kg/m^2^)  Overweight (25-29.9 kg/m^2^)  Obese (≥30.0 kg/m^2^) | 48 (2.0%)  1404 (59.8%)  594 (25.6%)  300 (12.8%) | 117 (2.4%)  2676 (54.8%)  1257 (25.7%)  836 (17.1%) | **<0.001** |
| Alcohol categories at FHRPC entry^2^:  Non-drinker  Low risk (≤14 units/wk)  Hazardous (14.1-35 units/wk)  Harmful (≥35 units/wk)  Missing | 1269 (42.5%)  1188 (39.8%)  465 (15.6%)  66 (2.2%)  n=295 | 1765 (34.9%)  2384 (47.2%)  784 (15.5%)  118 (2.3%)  n= 2586 | **<0.001** |
| Alcohol units per week (excluding categories of non-drinkers and missing)^3^ | 9.5 (5.0-16.0) | 9.0 (4.0-15.0) | **0.006** |
| Smoking status^2^:  Non-smoker  Former smoker  Current smoker  Missing | 1216 (54.0%)  449 (19.9%)  588 (26.1%)  n=1,030 | 2395 (52.5%)  1021 (22.4%)  1144 (25.1%)  n=3077 | 0.066 |
| Pack-years (former and current smokers only)^3^  Missing | 7.5 (2.8-15.0)  n=68 | 7.5 (2.5-15.0)  n=161 | 0.638 |

^1^ mean (SD) and independent samples t-test, ^2^ n (%) and Pearson Chi-Squared, ^3^ median (IQR: 25^th^ and 75^th^ percentiles) and Independent samples Mann-Whitney U Test

**Supplementary Table 3: BMI category transitions between time points**

Supplementary Table 3a: BMI category transitions between FHRPC entry and FHRisk completion (n=2,243 with data at both time points)

|  |  | | BMI category at FHRisk completion | | | |
| --- | --- | --- | --- | --- | --- | --- |
|  |  |  | Underweight | Healthy weight | Overweight | Obese |
| BMI category at FHRPC entry | | Underweight | 14 (29.2%) | 33 (68.8%) | 1 (2.1%) | 0 (0.0%) |
|  |  | Healthy weight | 15 (1.1%) | 949 (71.4%) | 325 (24.5%) | 40 (3.0%) |
|  |  | Overweight | 0 (0.0%) | 63 (11.3%) | 324 (58.3%) | 169 (30.4%) |
|  |  | Obese | 0 (0.0%) | 2 (0.7%) | 32 (11.5%) | 244 (87.8%) |

Supplementary Table 3b: BMI category transitions between age 20 and FHRPC entry (n=2,407 with data at both time points)

|  | |  | BMI category at FHRPC entry | | | |
| --- | --- | --- | --- | --- | --- | --- |
|  |  |  | Underweight | Healthy weight | Overweight | Obese |
| BMI category at age 20 | Underweight | | 22 (12.8%) | 134 (77.9%) | 14 (8.1%) | 2 (1.2%) |
|  | Healthy weight | | 21 (1.1%) | 1264 (67.1%) | 459 (24.4%) | 139 (7.4%) |
|  | Overweight | | 0 (0.0%) | 42 (17.9%) | 100 (42.6%) | 93 (39.6%) |
|  | Obese | | 0 (0.0%) | 3 (3.7%) | 17 (20.7%) | 62 (75.6%) |

Supplementary Table 3c: BMI category transitions between age 20 and FHRisk completion (n=2,883 with data at both time points)

|  |  | | BMI category at FHRisk completion | | | |
| --- | --- | --- | --- | --- | --- | --- |
|  |  |  | Underweight | Healthy weight | Overweight | Obese |
| BMI category age 20 | | Underweight | 18 (8.5%) | 153 (72.2%) | 30 (14.2%) | 11 (5.2%) |
|  |  | Healthy weight | 19 (0.8%) | 1178 (52.1%) | 755 (33.4%) | 309 (13.7%) |
|  |  | Overweight | 0 (0.0%) | 34 (10.9%) | 96 (30.9%) | 181 (58.2%) |
|  |  | Obese | 0 (0.0%) | 2 (2.0%) | 15 (15.3%) | 81 (82.7%) |

**Supplementary Table 4: Weight change percentage, and weight change per year**

|  | FHRPC entry to FHRisk  (mean 11.9 ±5.6 years) | | Age 20 to FHRPC entry  (mean 20.1 ±8.5 years) | | Age 20 to FHRisk  (mean 31.1 ±10.4 years) | |
| --- | --- | --- | --- | --- | --- | --- |
|  | Percentage weight change | Weight change per year (kg) | Percentage weight change | Weight change per year (kg) | Percentage weight change | Weight change per year (kg) |
| N* | 2212 | 2212 | 2375 | 2375 | 2886 | 2886 |
| Median (25^th^ -75^th^ percentile) | 4.5 (0.0-12.5) | 0.25 (0.00-0.68) | 9.7 (1.4-20.6) | 0.28 (0.04-0.60) | 15.8 (6.2-28.7) | 0.30 (0.12-0.55) |

*using all available valid data

**Supplementary Table 5: Comparison of weight changes over the three joining periods: ANCOVA (using all available data)**

|  | | | Parameter estimates | | | | | Estimated marginal means | | |
| --- | --- | --- | --- | --- | --- | --- | --- | --- | --- | --- |
|  | n | period | B  (Regression Coefficient) | Std Error | Sig. | 95% Confidence Interval | | Mean Change (kg) | 95% Confidence Interval | |
|  |  |  |  |  |  | Lower Bound | Upper Bound |  | Lower Bound | Upper Bound |
| Weight change FHRPC entry to completing FHRisk questionnnaire^2^ | 1096 | 1989-1998 | 2.2 | 1.2 | 0.06 | -0.1 | 4.6 | 4.8 | 4.0 | 5.6 |
|  | 877 | 1999-2008 | 0.9 | 0.7 | 0.21 | -0.5 | 2.4 | 3.5 | 2.7 | 4.2 |
|  | 171 | 2009-2018 | Ref | | | | | 2.5 | 0.8 | 4.3 |
| Weight change age 20 to FHRPC entry^1^ | 1176 | 1989-1998 | -3.4 | 0.7 | **<0.001** | -4.7 | -2.2 | 6.0 | 5.5 | 6.6 |
|  | 846 | 1999-2008 | -1.5 | 0.7 | **0.03** | -2.8 | -0.1 | 8.0 | 7.4 | 8.7 |
|  | 272 | 2009-2018 | Ref | | | | | 9.5 | 8.3 | 10.6 |
| Weight change age 20 to completing FHRisk questionnaire^3^ | 1173 | 1989-1998 | 0.2 | 0.8 | 0.82 | -1.4 | 1.7 | 10.9 | 10.2 | 11.6 |
|  | 1276 | 1999-2008 | 0.5 | 0.7 | 0.53 | -0.9 | 1.8 | 11.2 | 10.5 | 11.8 |
|  | 337 | 2009-2018 | Ref | | | | | 10.7 | 9.4 | 12.0 |

1 Weight change age 20 to FHRPC entry (kg): Dependent variable: weight change age 20 to FHRPC entry / Fixed factor: period group / Covariates: weight age 20, duration between age 20 and FHRPC entry, IMD decile

2 Weight change FHRPC entry to FHRisk completion (kg): Dependent variable: weight change FHRPC entry to FHRisk completion / Fixed factor: period group / Covariates: weight at FHRPC entry, duration between FHRPC entry and FHRisk completion, IMD decile

3 Weight change age 20 to FHRisk completion: Dependent variable: weight change age 20 to FHRisk completion / Fixed factor: period group / Covariates: weight at age 20, duration between age 20 and FHRisk completion, IMD decile

**Supplementary Table 6: BMI at each point for those with no missing BMI data (n=2,075), n (%)**

|  | **Age 20** | **FHRPC entry**  **(mean age 39.6 ±8.8)** | **FHRisk**  **(mean age 51.4 ±10.4)** |
| --- | --- | --- | --- |
| Underweight (<18 kg/m^2^) | 149 (7.2) | 45 (2.2) | 27 (1.3) |
| Healthy weight (18–24.9 kg/m^2^) | 1,646 (79.3) | 1,269 (61.2) | 994 (47.9) |
| Overweight (25.0–29.9 kg/m^2^) | 206 (9.9) | 509 (24.5) | 634 (30.6) |
| Obese (≥30.0 kg/m^2^) | 74 (3.6) | 252 (12.1) | 420 (20.2) |
|  |  | p=<0.01* | p=<0.01*  p=<0.01** |

* McNemar-Bowker Test compared to BMI categories at age 20

** McNemar-Bowker Test compared to BMI categories at FHRPC entry
